# 

**Published:** 1865-09

**Authors:** 


### CONTENTS.

ORIGINAL ESSAYS AND COMMUNICATIONS.

Professional Etiquette.... 349
Lancing the Gums of Infants.... 353
Lancing the Gums in Extraction.... 355

PROCEEDINGS OF SOCIETIES.

Proceedings of the Iowa State Dental Society.... 358
Western Dental Society.... 386

SELECTIONS.

Wounds on the Face.... 391

EDITORIAL.

A New Dental College.... 392
Personal.... 393
The Steam Gauge for Vulcanizing.... 393
The Iowa State Dental Society.... 394

